# Revised domain structure of ulvan lyase and characterization of the first ulvan binding domain

**DOI:** 10.1038/srep44115

**Published:** 2017-03-22

**Authors:** Rebecca L. J. Melcher, Marten Neumann, Juan Pablo Fuenzalida Werner, Franziska Gröhn, Bruno M. Moerschbacher

**Affiliations:** 1 University of Münster, Institute for Biology and Biotechnology of Plants, Schlossplatz 8, D-48143 Münster, Germany; 2 Friedrich-Alexander-University Erlangen-Nürnberg, Department of Chemistry and Pharmacy, Interdisciplinary Center for Molecular Materials, Egerlandstraße 3, D-91058 Erlangen, Germany

## Abstract

Biomass waste products from green algae have recently been given new life, as these polysaccharides have potential applications in industry, agriculture, and medicine. One such polysaccharide group called ulvans displays many different, potentially useful properties that arise from their structural versatility. Hence, performing structural analyses on ulvan is crucial for future applications. However, chemical reaction–based analysis methods cannot fully characterize ulvan and tend to alter its structure. Thus, better methods require well-characterized ulvan-degrading enzymes. Therefore, we analysed a previously sequenced ulvan lyase (Genebank^TM^ reference number JN104480) and characterized its domains. We suggest that the enzyme consists of a shorter than previously described catalytic domain, a newly identified substrate binding domain, and a C-terminal type 9 secretion system signal peptide. By separately expressing the two domains in *E. coli*, we confirmed that the binding domain is ulvan specific, having higher affinity for ulvan than most lectins for their ligands (affinity constant: 10^5^ M^−1^). To our knowledge, this is the first description of an ulvan-binding domain. Overall, identifying this new binding domain is one step towards engineering ulvan enzymes that can be used to characterize ulvan, e.g. through enzymatic/mass spectrometric fingerprinting analyses, and help unlock its full potential.

The green algal biopolymer ulvan is an underexploited biomass in spite of its promising applications in the food industry, e.g. as a dietary fibre or as an antioxidant^1^, and in the chemical industry as a source for rare sugars. Additionally, its anti-viral, anti-coagulant, and anti-proliferative activities towards cancer cells^2^ and its immune stimulating properties^3^ make ulvan interesting for pharmaceutical and medical applications. It can be used to build biomedically relevant structures such as nano-fibres^4^, membranes^5^, particles^6^, and hydrogels^7^. Furthermore, it shows potential for agricultural applications, as it has been shown to be an activator of plant defence^8^ and an inducer of plant resistance^9^. The individual composition and structure of ulvan polymers may differ from species to species, and they are further influenced by external factors like growth conditions or harvesting season, and even by the method of extraction^10^,^11^.

Ulvans are sulphated hetero-polysaccharides derived from green macro algae of the genus *Ulva*^9^,^12^. The backbone of these polysaccharides mainly consists of rhamnose, glucuronic-acid, iduronic-acid, and xylose, and it can be sulphated (up to 30%) at rhamnose (C3) and xylose (C2)^12^ units. Their main building blocks are ulvanobiuronic acids, i.e. glucuronic acid or iduronic acid α or b (1, 4) linked to sulphated rhamnose, and ulvanobioses, i.e. (sulphated) xylose α or b (1, 4) linked to sulphated rhamnose^13^, but many other building blocks have been described^12^.

Because their properties strongly depend on molecular weight^14^, sulphate content^15^, and monosaccharide composition^16^, detailed structural analysis of ulvan polymers is crucial. However, with the different characteristics of each monomer unit, chemical methods are not trivial and tend to change the composition^17^,^18^. In the future, enzymes acting on ulvan might become more powerful analytical tools since they offer a more specific approach, which may eventually allow for enzymatic fingerprinting^19^. As such, well-characterized enzymes may refine the understanding of ulvan structure and might eventually make ulvan sequencing possible.

A major breakthrough in ulvan structure analysis occurred with the discovery of the first endo-ulvan lyase^20^. Since then, only a few ulvan-degrading enzymes have been found, e.g. in marine bacteria. Besides two ulvan lyases, which cleave the polymer between sulphated rhamnose and glucuronic acid units^20^,^21^, other enzymes that work on ulvan oligomers are a ß-glucuronidase^22^, a glucuronan lyase^23^, and an ulvan hydrolase^21^,^24^. Recently four more ulvan lyases were found in the ulvan-degrading species *Alteromonadales*^25^. Interestingly, the first detailed analysis of an ulvan lyase^21^ revealed that it belongs to a new lyase family. Through investigating this ulvan lyase further, first by recombinantly expressing it in *E. coli,* we gained insight into the domain structure of the enzyme and discovered, among other things, the first ulvan binding domain. To prove the functionality of this domain, we expressed it separately from the catalytic lyase domain and investigated its binding specificity and affinities in different assays.

## Results

### Domain structure

**Figure 1:**
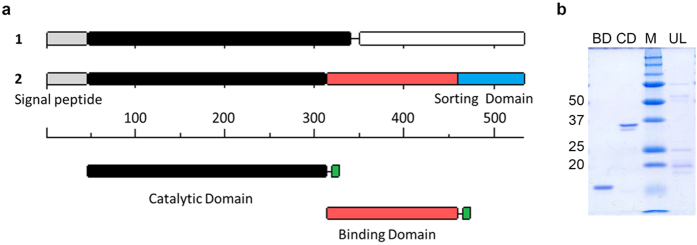
Revised structure of *N. ulvanivorans* lyase. (**a**) Domain structure as described by Collén 2011 (1) with (from left to right) Signal peptide (grey), catalytic domain (black) and domain of unknown function (white), in contrast to our proposed domain structure (2) with same signal peptide (grey), shorter catalytic domain (black) and a binding domain (blue). Shown is a marker with amino acids to indicate the length of domains, as well as the constructs for catalytic domain (black) and binding domain (blue) with a Strep-tag II (green). (**b**): SDS-PAGE gel of recombinantly expressed domains, with binding domain (BD), catalytic domain (CD) marker (M) and full-length construct (UL).

To overcome previously described^21^ problems associated with recombinantly expressing the recently discovered ulvan lyase from *Nonlabens ulvanivorans* (previously *Percicivirga ulvanivorans*)^21^,^26^ in *E. coli,* we carefully re-examined its domain structure. Previously, the enzyme was described as having two domains: a catalytic domain with an N-terminal signal peptide and a second domain of unknown function, separated by a low complexity region^21^. Bioinformatic analyses showed the C-terminal domain to have low similarity (E-value 1.18E-7) to a ricin/lectin-like domain, and analyses also showed the presence of a C-terminal signal peptide for the Por-secretion system (E-value 4.4E-18). We thus suggest the domain structure to be as follows (Fig. 1a): The N-terminal part consisting of the already described signal peptide and the catalytic module, slightly shorter than previously described^21^, followed by a substrate-binding domain (including the low complexity region) and a C-terminal-Por secretion signal.

### Recombinant expression

To validate this structure and to confirm the function of the binding domain, we established two deletion constructs containing either only the binding domain or only the catalytic domain, without the N- and C-terminal signal sequences (Fig. 1a). We were able to purify the catalytic domain, achieving the expected size of 31 kDa, as well as the binding domain, with its expected size of 18 kDa (Fig. 1b). The full-length construct of ca. 47 kDa was produced in small amounts only and always contained a number of N-terminal degradation products. Both the catalytic domain and the full-length protein showed a double band; the lower band disappeared when the protein was pre-incubated with DTT, indicating the presence of a disulphide bond (Supplements).

### Influence on lyase activity

**Figure 2:**
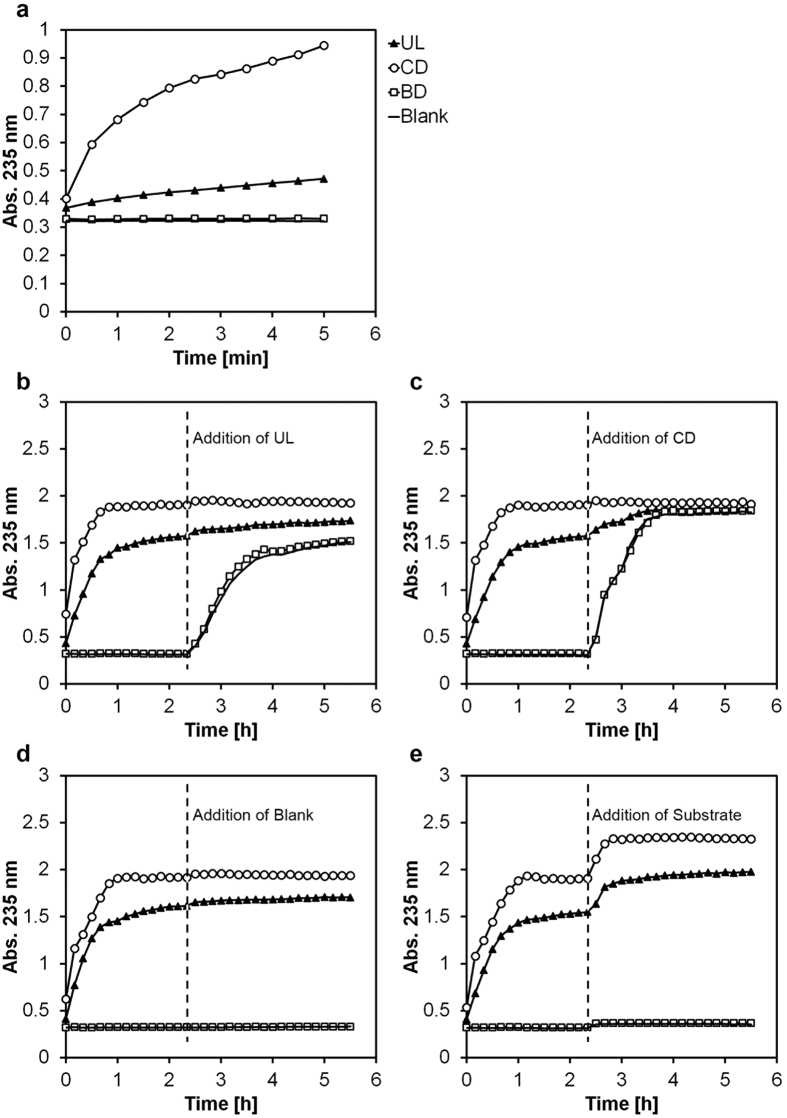
Ulvan lyase activity. The formation of double bonds was measured at 235 nm in UV 96-well plates using a SpectraMax^®^ M2 Multi-Mode Microplate Reader at 30 °C. The assay was performed with 198 μl ulvan buffer (1 g/l ulvan, 100 mM Tris-HCl, pH 8.5, 200 mM NaCl). Two μl of enzyme (10 pmol) was added and the measurement started immediately. Lyase activity of catalytic domain (CD), binding domain (BD) and full length construct (UL) (**a**). Lyase activity over 6 h, with additional addition of the full-length construct (UL) (**b**), catalytic domain (CD) (**c**), water (blank) (**d**) or ulvan (substrate; 20 μg/well) (**e**) after 2.5 h.

We tested all three constructs for catalytic activity towards polymeric ulvan by measuring the appearance of double bonds at 235 nm, which arises due to the enzyme’s lytic rather than hydrolytic mode of action (Fig. 2a). We observed lyase activity for the full-length protein and for the catalytic domain, with the latter being more active than the former; as expected, the binding domain did not show catalytic activity. The catalytic domain reached a higher final substrate degradation rate than the full-length protein. Reciprocal enzyme additions after reaching the end point of the reaction confirmed that the catalytic domain alone could further degrade the product of the full-length enzyme, but not vice versa (Fig. 2b–e). TLC analysis showed the appearance of oligomeric products upon incubation of ulvan with the full-length construct and the catalytic domain alone, but not with the binding domain, confirming the binding domain’s lack of lytic or hydrolytic activity (Supplements).

### Binding ability, substrate specificity and substrate affinity

**Figure 3:**
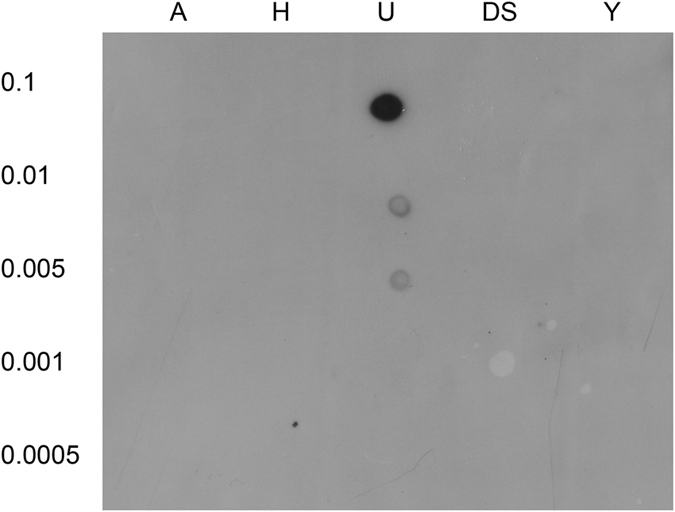
Dot Blot Binding assay. Solubilized Polysaccharides (Alginate (A), Heparin (H), Ulvan (U), Dextransulphate (DS) and Iota Carrageenan (Y)) in different concentrations (0.1–0.0005 μg) were immobilized on a SuPerCharge-Nylon Membrane by baking for 30 minutes at 70 °C. Afterwards the membrane was washed and blocked using 3% (w/v) milk powder for 1 h at room temperature, followed by severe washing. The membrane was incubated with BD for 30 min followed by washing steps and incubation with *Strep-*Tactin–horseradish peroxidase (HRP) conjugate. The signal was detected by chemiluminescence.

**Figure 4:**
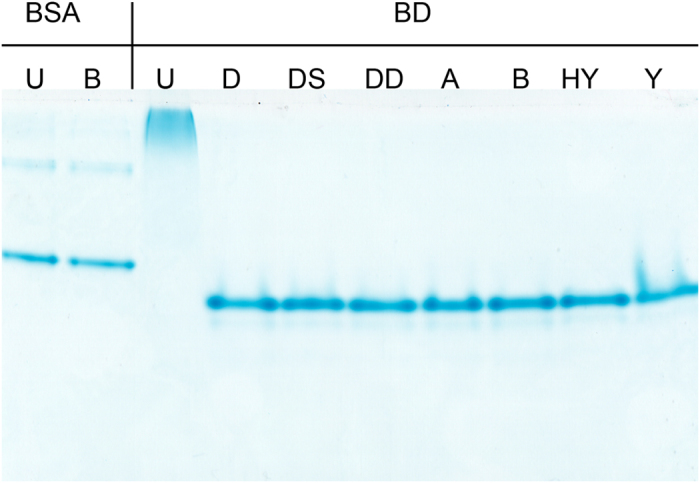
Proteins- substrate interactions Gel shift assay. Proteins (Bovine serum albumin (BSA) as a negative control and the binding domain (BD)) (1.5 μg) were incubated with polymers (6 μg each of ulvan (U), dextran (D), dextransulphate (DS), DEAE dextran (DD), alginate (A), heparin (HY) and iota carrageenan (Y)) for 30 min at 30 °C. Negative controls were incubated with buffer instead. Analysis was performed using polyacrylamide gel electrophoresis without addition of SDS.

We then tested the binding domain for its ulvan-binding ability and ligand specificity in a dot blot assay (Fig. 3). While the domain bound strongly to ulvan, it did not bind to other polymers (alginate, heparin, dextran sulphate, iota carrageenan) that are structurally similar to ulvan. The same specificity was seen in a gel-shift assay, where the migration of the binding domain was only slowed down by ulvan (Fig. 4).

**Figure 5:**
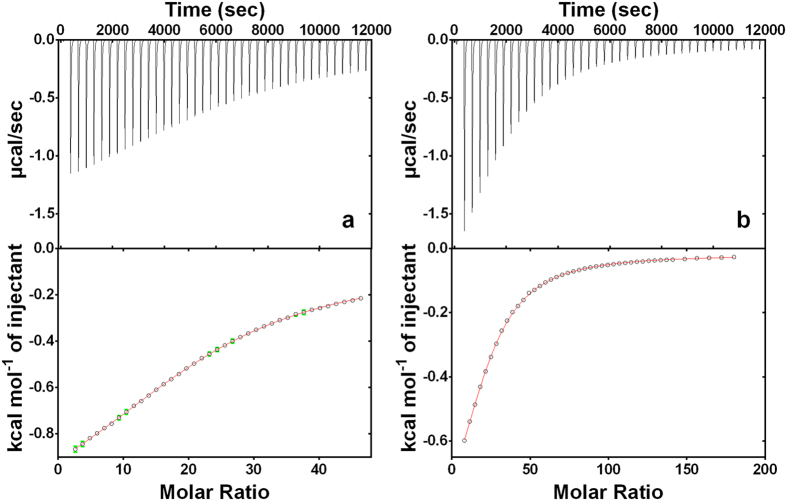
Calorimetric titrations for ulvan binding domain to ulvan polymer. ITC profiles corresponding to 2546 μM of ulvan titrated into 24.6 μM of ulvan binding domain (**a**); 5155 μM of ulvan titrated into 16.4 μM of ulvan binding domain (**b**). The solid lines in the bottom panels represent the best curve fits to the experimental data, using the 1:1 model from SEDPHAT.

To further confirm and quantify the interaction between the ulvan-binding domain and ulvan, ITC experiments were performed. Figure 5a,b show two independent titrations made with two different production batches of the binding domain at different concentrations of protein and ulvan. As the thermogram of titration a clearly shows, the end of the reaction was not reached in this experiment. Therefore, titration b was performed with a lower amount of protein in the cell and a higher ligand concentration in the syringe. The fitting of both titrations gave similar results, and the interaction can be globally fitted. The thermodynamic parameters show an enthalpy-driven association with a significant entropic cost, an affinity constant of 10^5^ M^−1^, and a stoichiometry of seven mono-sulphated ulvan units per binding protein (Table 1).

## Discussion

We propose an improved domain structure of the ulvan lyase with an N-terminal signal peptide followed by the catalytic domain, an ulvan binding domain, and a C-terminal Por secretion signal. This improved domain annotation allowed us to recombinantly express the catalytic and the substrate-binding domains in *E. coli,* yielding recombinant proteins for functional studies. While only the N-terminal domain was catalytically active, the C-terminal domain specifically bound to ulvan. Similar to the earlier study^21^, the full-length protein was difficult to express, yielding low amounts of recombinant enzyme and a number of degradation products. However, removal of the C-terminal Por secretion signal in our study led to sufficient production of recombinant protein for functional studies.

While we did not experimentally validate the putative signal peptide of the Por secretion system, a number of cues suggest this function. First, *N. ulvanivorans* secretes this enzyme into the medium, and members of the *Bacteroides* seem to almost exclusively use the Por secretion system^27^. Second, the size of the secreted enzyme was reported as either 30 or 46 kDa depending on growth conditions^21^, but never 56 kDa, which would be the predicted size based on the amino acid sequence derived from the gene when only the N-terminal signal peptide is removed. Given that the Por signal peptide is cleaved off during secretion^28^,^29^, the observed sizes of 30 and 46 kDa are in perfect agreement with the domain structure proposed here, representing the catalytic domain alone and the full-length mature protein consisting of the catalytic and binding domains, respectively. Third, Por-mediated secretion of a two-domain chitinase from *Flavobacterium johnsoniae* has also been reported to lead to proteolytic cleavage between the domains^30^. Last, peptides corresponding to the catalytic domain have been identified in both the 30 and the 46 kDa proteins, while peptides corresponding to the binding domain were present only in the 46 kDa protein, and peptides corresponding to the C-terminal Por signal peptide were absent from both proteins^21^.

Substrate-binding domains are often encountered in hydrolytic enzymes that act on complex, poorly soluble substrates, such as structural polysaccharides in fungal, plant, or algal cell walls. Presumably, these domains serve to anchor the enzyme at the substrate, thus improving catalysis, but often at the cost of turnover number if the substrate is presented in a soluble form. This also seems to be the case here: towards the soluble substrate ulvan, the catalytic domain alone seems more active than the full-length enzyme with the binding domain attached. Possibly, the binding domain helps the enzyme to act on ulvan in its insoluble form embedded in the algal cell wall. Interestingly, the catalytic domain alone also led to a more extensive final depolymerisation than the full-length enzyme, and reciprocal complementation experiments showed that the catalytic domain was able to further degrade the final product of the full-length enzyme, but not vice versa. This indicates that there are additional cleavage sites for the catalytic domain that are not accessible to the full-length enzyme.

Dot blot analyses and gel retardation assays showed that the binding of the C-terminal domain is specific for ulvan. To our knowledge, this is the first description of an ulvan-binding domain. Its amino acid composition predicts a net charge of −25 at pH 8.5, the pH optimum of the enzyme, indicating that the binding is not electrostatic in nature. To quantitatively analyse the interaction between the binding domain and its ligand ulvan, we performed ITC analyses, which measure the amount of heat released or absorbed upon a molecular interaction. This experimental information can be further processed to obtain a complete thermodynamic profile - i.e., binding constant (K), enthalpy change (ΔH), entropy change (ΔS), Gibbs free energy change (ΔG) - of the molecular interaction^31^. We found that the interaction was enthalpy driven, possibly reflecting the occurrence of multiple contact points at the binding site made possible by hydrogen bonds, van der Waals interactions, protonation, or ligand-induced conformational changes in the protein’s binding pocket^32^,^33^. Complementarily, the entropic cost may reflect the enzyme’s need to structurally fix the highly flexible ulvan molecule, which is achieved by locking the polysaccharide in a particular conformation and decreases its rotational and translational freedom^33^,^34^. The affinity between proteins and carbohydrates is typically rather low, usually in the millimolar range^32^. However, ITC showed a stronger interaction between the ulvan-binding domain and ulvan, probably affected by the polymer structure in solution, confirming the specificity of the interaction. Further experiments varying the type of ulvan polymer or the conditions (ionic strength and temperature) could give more hints about the binding mechanism that governs this interaction.

The inflection point of the enthalpogram shows an N value of 7 so one protein binds to seven monosulphated units of ulvan. According to the classic interpretation, this would mean that one protein has seven equal binding sites. As this is unlikely, we suggest an approach according to Zhao and co-workers, where the inflection point in the ITC profiles does not represent the N value in a known 1:1 binding process^35^. Here a different parameter is extracted from the ITC profiles called the “incompetent fraction” (which is constrained between 0 and 1)^36^. This interpretation leads to an inactive fraction of ulvan around 86%, suggesting only 14% of all monosulfated ulvan units injected bind to the protein, which is equal to one binding event every seven units. With on average one sulphate group per 2.5 monosaccharide residues^8^, this indicates one binding event every 17 monosaccharide residues. With a molecular weight of 18 kDa, the ulvan-binding domain can be estimated to have a diameter of ca. 2–4 nm^37^, corresponding to ca. 2–4 monosaccharide residues, in keeping with the observation that ricin/lectin-like domains usually have a small binding pocket accommodating mono-, di- and trisaccharides^38^. We thus assume that a small binding site of ca. 2–4 monosaccharide units occurs every ca. 17 units in the chain. Given the complex structure of ulvan, this assumption seems plausible.

Upon re-examination of the domain structure of the first ulvan lyase described^21^, we found that its C-terminal ‘domain of unknown function’ is a substrate-binding domain with specificity and high affinity for ulvan. As such, we successfully identified, for the first time, an ulvan-binding domain. This domain seems to not only influence the activity of the catalytic domain quantitatively, but also qualitatively, leading to more productive binding and different cleavage sites. Once we understand this process better, it will offer an additional tool to study the structure of ulvan. Also, the recombinant ulvan-binding protein has other potential uses, such as producing a fusion protein with GFP which can be used as an ulvan-specific lectin, or producing an ulvan affinity column to purify specific ulvans. It could also be used to develop and functionalize ulvan-based materials, because polysaccharide-binding proteins offer a promising route to attach functional proteins to polysaccharides. For example, an ulvan-binding protein could be used to attach a fluorescent protein to an ulvan-based membrane or nanoparticle to obtain light-traceable materials, as we have already shown for chitosan^39^. Overall, the possibilities for using this newly identified ulvan-binding domain, either for characterizing ulvan or for manipulating it, are potentially quite powerful.

## Materials and Methods

### Ulvan

Ulvan was extracted from *Ulva fasciata* as previously described^8^ and its molecular weight of about ~600,000 g/mol was confirmed by high-performance size exclusion chromatography (Agilent Technologies, Santa Clara, USA.) on three PSS^®^ Suprema columns (one 100 Å guard column and two 3000 Å columns with an internal diameter of 8 mm) coupled to a refractive index detector (Agilent series 1200 RID). The molecular weight was determined by calibration using a series of pullulans (PSS, Mainz, Germany). The validity of calibration for negatively-charged polysaccharides was confirmed using dextran sulphates (Sigma-Aldrich, Taufkirchen, Germany).

### Bioinformatical tools

For investigation of the domain structure, InterPro^40^ was used. Results were then verified with comparison to the Superfamily Database^41^ and the HMMscan-tool^42^.

### Bacterial strain, plasmid and culture medium

Recombinant plasmids were kept in *Escherichia coli* DH5α, and for recombinant protein expression, *E. coli* Rosetta 2(DE3)(pLysSRARE2) was used (Merck, Darmstadt, Germany). Plasmids were sequenced at MWG-Biotech AG (Ebersberg, Germany). The pET-22b(+) vector (Merck) was used for all constructs. Both *E. coli* stains were grown in LB at 37 °C. For the selection of transformants 100 μg/ml ampicillin *(E. col*i DH5α) or 100 μg/ml ampicillin plus 34 μg/ml chloramphenicol (*E. coli* Rosetta 2(DE3)(pLysSRARE2)) was added. For optimal protein expression autoinduction was performed according to ref. 43 as follows: Addition of autoinduction solutions M (50x stock: 1.25 MNa_2_HPO_4_, 1.25 M KH_2_PO_4_, 2.5 M NH_4_Cl, 0.25 M Na_2_SO_4_), 5052 (50x stock: 25% [v/v] glycerol, 2.5% [w/v] D-glucose, 10% [w/v] -lactose monohydrate) and 2 mM MgSO_4_ induced the cells for expression of the target protein. To obtain sufficient amounts of soluble protein, it was obligatory to grow the cultures for 4 h at 37 °C (180 rpm), 18 h at 20 °C (180 rpm) and 4 h at 20 °C (120 rpm). After addition of IPTG to increase protein yield (0.1 mM for CD; 0.3 mM for BD) cells were grown for 3.5 h before harvest. Stocks were prepared for long-time storage with 30% (v/v) glycerol and kept at −70 °C.

### Cloning

Base pairs 142 to 1602 of the *N. ulvanivorans* ulvan lyase coding gene sequence (JN104480.1 (GenBank)) were used to generate an artificial gene sequence that was codon optimized for expression in *E. coli* (Geneart, Regensburg, Germany). Due to its 5 prime sequence truncation, the encoded protein does not contain the N-terminal signal peptide identified by Nyvalle-Collén and co-workers^21^. This “full-length” (UL) sequence was cloned into the pET-22b(+) expression vector (Merck) by using a MscI/EcorI-cloning strategy resulting in the pET22b(+)::FL-StrepIIC plasmid.For expression and secretion into the periplasm via pelB leader sequence an NdeI/EcorI-cloning approach was used, which led to the pET22b(+)::FL-StrepIIC_(exp.)_ vector. The BD- and CD-construct coding plasmids pET22b(+)::BD-StrepIIC and pET22b(+)::CD-StrepIIC_(exp.)_ were derived from these vectors by PCR. The necessary deletion was performed by using 5′ phosphorylated “back to back” primer pairs (Supplemental materials).

### Expression and purification

Both domains were synthesized in *E. coli* Rosetta 2(DE3)(pLysSRARE2). Harvested cells were resuspended in 40 mM triethanolamine (pH 8) buffer containing 400 mM NaCl and frozen at −20 °C for at least 24 h. Afterwards, cells suspensions were incubated for 30 min at 37 °C. Simultaneously, 30 mM triethanolamine (pH 8), 30 mM NaCl, 6 mM MgCl_2_, 1 mM phenylmethylsulfonyl fluoride, and 10 U/ml benzonase were added together with approximately 4000 U/mL lysozyme. The cells were disrupted by brief sonication and the crude extracts were obtained by centrifugation. The *Strep*-tag II containing target proteins were purified from the crude extract by affinity chromatography using *Strep*-Tactin columns (*Strep*-Tactin superflow Plus, 1-ml bed volume; Qiagen, Hilden, Germany) according to supplier’s instruction and concentrated in ultrafiltration devices (Sartorius Stedim Biotech, Göttingen, Germany) with a molecular mass cut-off at 10 kDa. For storage at 4 °C, 10% (v/v) glycerol was added. The protein concentration was determined after Bradford^44^ with bovine serum albumin (BSA) as the standard and the purity was subsequently analysed by SDS-polyacrylamide gel electrophoresis^45^.

### Enzyme activity

The formation of double bonds was measured at 235 nm to follow the lyase activity as described before^21^ with minor modifications. Measurement occurred in UV 96-well plates (Thermo Fischer, Waltham, USA) using a SpectraMax^®^ M2 Multi-Mode Microplate Reader (Molecular Devices Corporation, Sunnyvale, USA) set at 30 °C. The assay was performed with 198 μg ulvan (1 g/l ulvan, 100 mM Tris-HCl, pH 8.5, 200 mM NaCl). Two μl of enzyme (10 pmol) was added and the measurement started immediately. Absorbance was recorded for 5 min every 30 seconds.

### Dot Blot Assay

The binding assay was performed as described before^46^ with minor modifications: Solubilized polysaccharides in different concentrations (2 μl each) were immobilized on a SuPerCharge-Nylon Membrane (Schleicher & Schuell BioScience, Dassel, Germany) by baking for 30 min at 70 °C. Afterwards, the membrane was washed with 1 × TBS buffer twice for 5 min and then blocked using 3% (w/v) milk powder in 1 × Tris-buffered saline (TBS) for 1 h at room temperature, followed by washing four times with 1 × TBS for 5 min. After washing, the membrane was incubated with BD for 30 min at room temperature. Washing steps were again continued with 1 × TBS four times for 5 min each, and then the membrane was incubated with *Strep*-Tactin–horseradish peroxidase (HRP) conjugate (IBA, Göttingen, Germany) and the signal was detected by chemiluminescence.

### Gel Shift Assay

Interaction of polymer and protein leads to complex formation that visibly influences behaviour in native PAGE (polyacrylamide gel electrophoresis^45^ without addition of SDS). Here 1.5 μg of protein were incubated with different polymers (6 μg) for 30 min at 30 °C. Negative controls were incubated with buffer instead. Analysis occurred via native PAGE.

### ITC

The interaction of the ulvan-binding domain with ulvan was quantified using isothermal titration calorimetry on a MicroCal VP-ITC instrument (MicroCal LLC, Northampton, MA, USA). Consecutive injections of 5 μL aliquots of ulvan solution were added with the help of a rotator stirrer-syringe into the calorimeter cell of 1.445 ml filled with ulvan-binding protein in a 0.1 M TRIS buffer at pH 8.5 containing 200 mM NaCl at 25 °C. To minimize the contribution of heat of dilution to the measured heat change, the protein and ulvan solutions were prepared in the same buffer. Injections were made at intervals of 4 min for all titrations. Control experiments were performed by injecting ulvan solution into the buffer solution in an identical manner, and the resulting heat changes were subtracted from the measured heats of binding. Since the first injection is often inaccurate, a 1 or 2 μl injection was added first and the resultant point was deleted before the remaining data were analysed. Integration of the injection peaks and construction of binding isotherms were performed by using the high-precision automated peak shape analysis software NITPIC^47^. The different titrations were analysed separately and globally in SEDPHAT by using the 1:1 association model^35^. Parameter precision was determined by using the surface projection method and a critical χ2 cut-off value based on Fisher statistics at a 68% confidence level. Ulvan concentration was calculated as the molar concentration of monosulphated ulvan units, i.e. based on the average MW of an ulvan section carrying a single sulphate group. Based on the composition of the ulvan used^8^, the average MW of a monosulphated ulvan unit was calculated as 508 g/mol, corresponding to one sulphate group per 2.5 monosaccharide units.

## Additional Information

**How to cite this article**: Melcher, R. *et al*. Revised domain structure of ulvan lyase and characterization of the first ulvan binding domain. *Sci. Rep.* **7**, 44115; doi: 10.1038/srep44115 (2017).

**Publisher's note:** Springer Nature remains neutral with regard to jurisdictional claims in published maps and institutional affiliations.

## Supplementary Material

Supplementary Information

## Figures and Tables

**Table 1:** Thermodynamic parameters obtained by isothermal titration.

	UBD (μM)	ulvan (μM)	LogKa	ΔH (kcal/mol)	TΔS (kcal/mol)	n	I.F. ulvan
Titration a	24.6	2546	4.69	−36.45	−30.05	7.18	0.860
Titration b	16.4	5155	5.03	−22.42	−15.56	7.00	0.857
Titration a + b	—	—	5.03	−22.25	−15.60	7.00	0.852

Calorimetry for A: 2546 μM of ulvan titrated into 24.6 μM of ulvan binding domain; B: 5155 μM of ulvan titrated into 16.4 μM of ulvan binding domain and a + b: results of the global analysis of both titrations simultaneously.

UBD: Ulvan Binding Domain

n: Number of monosulphated polysaccharide units per UBD

I.F.: Incompetent fraction.
