# Supplementary material for: Revised domain structure of ulvan lyase and characterization of the first ulvan binding domain
Source: Sci Rep. 2017 Mar 22;7:44115. doi: 10.1038/srep44115 (PMC5361163; doi:10.1038/srep44115)
Supplement: Supplementary Information [file srep44115-s1.pdf]

Supplementary data

Revised domain structure of ulvan lyase and characterization of the first ulvan binding domain

Rebecca Melcher[1], Marten Neumann[1], Juan Pablo Fuenzalida Werner[2], Franziska Gröhn[2] and Bruno M. Moerschbacher*[1]

1 University of Münster, Institute for Biology and Biotechnology of Plants, Schlossplatz 8, D-48143 Münster, Germany

2 Friedrich-Alexander-University Erlangen-Nürnberg, Department of Chemistry and Pharmacy, Interdisciplinary Center for Molecular Materials, Egerlandstraße 3, D-91058 Erlangen, Germany

*Corresponding Author: B. M. Moerschbacher; Institute for Biology and Biotechnology of Plants; moersch@uni-muenster.de

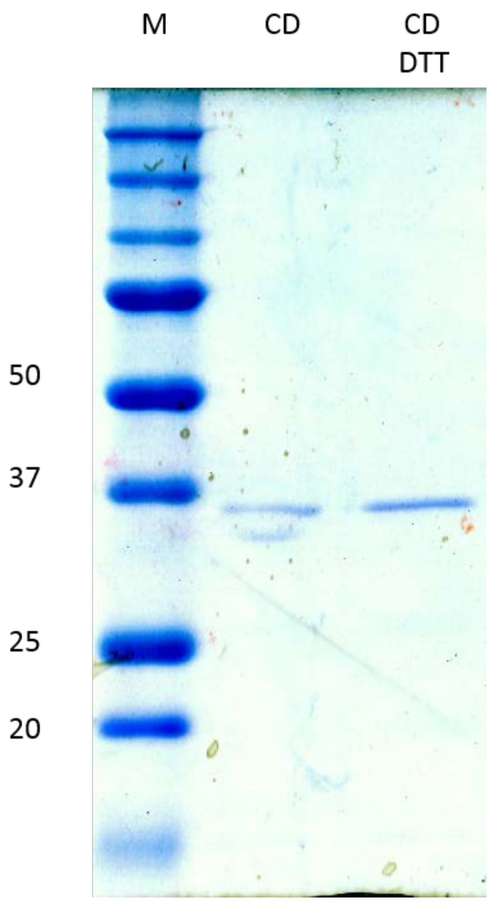

Supplement 1: SDS page of catalytic domain with DTT incubation
Catalytic Domain (CD) with and without preincubation with DTT on a SDS page

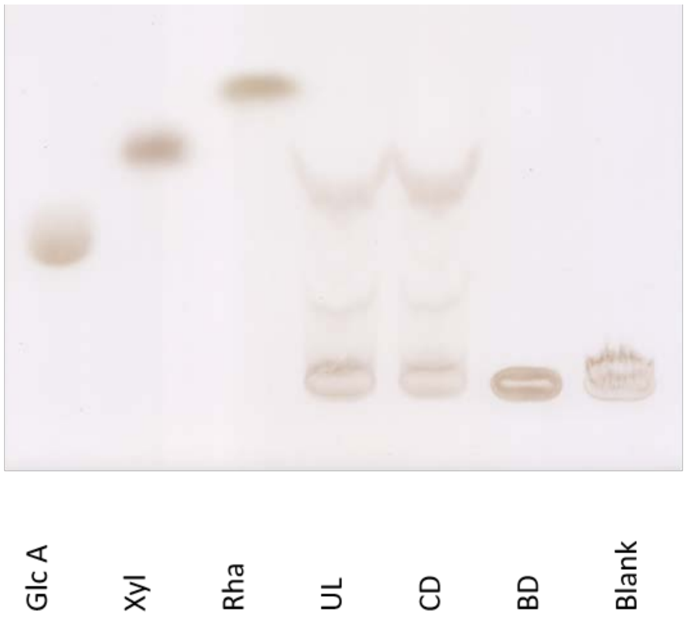

Supplement 2: TLC of Ulvan
Polymer was preincubated for 6h with full length construct (UL), catalytic domain (CD), binding domain (BD) and water (blank). Glucuronic acid (Glc A), Xylose (Xyl) and Rhamnos (Rha) were used as standard.

Supplement 3: Primers

Name	Sequence	Temperatur	Note
CtermSigPepdel	ACCAACGGACGCAATTTGCTGAC	62.4 °C	5‘-phosphorylation
ULopfull/catFw1	GATAATGGAACTGCCAGAGC	57.3 °C	5‘-phosphorylation
ULopfull/bindRv1	GCATCTACCAGTGGCACG	58.2 °C	5‘-phosphorylation
UL(op)_cat_rev2	GAATTCGAGCTCCGTCGACAATG	62;4 °C	5‘-phosphorylation
UL(op)bindingfw2	CATATGTATATCTCCTTCTTAAAGT	54.8 °C	5‘-phosphorylation
BD2(op)_rev	AGCACCATTCCGGCAGCTGA	61.4 °C	5‘-phosphorylation
CD(secr.)postm	ATTGTCAACCACTTCAACTT	51.1°C	5‘-phosphorylation
Forward Ul/Bd	CG<u>GAATTC</u>GTTGACGATCAG	58.4 °C	*Eco*RI-sequence
Forward Cd	CG<u>GAATTC</u>GATAATGGAACTGC	57;3 °C	*Eco*RI-sequence
UL/CD Rev.	CCATGGCCCCGGAC	50.0 °C	
BD Rev	CCATGGCATCTACCAGTG	59.0 °C	
T7	TAATACGACTCACTATAGGG	53.2 °C	
T7term	CTAGTTATTGCTCAGCGGT	50.2 °C	
